# Supplementary material for: In vivo lentiviral vector gene therapy to cure hereditary tyrosinemia type 1 and prevent development of precancerous and cancerous lesions
Source: Nat Commun. 2022 Aug 25;13:5012. doi: 10.1038/s41467-022-32576-7 (PMC9411607; doi:10.1038/s41467-022-32576-7)
Supplement: Supplementary file 1 — Supplementary Information [file 41467_2022_32576_MOESM1_ESM.pdf]

Supplemental Data

Supplemental Figure 1. Further acute inflammatory markers and vital signs after LV administration. (a) Further acute inflammatory response (IL-8 and platelets) after systemic LV-FAH administration compared to reference portal vein LV-GFP delivery. (b) Further vital signs (temperature) after portal LV-FAH administration. (c) Vital signs after systemic LV-FAH and LV-GFP delivery compared to portal LV-GFP delivery. Blood pressure was artificially maintained with pressors in both systemic delivery animals.

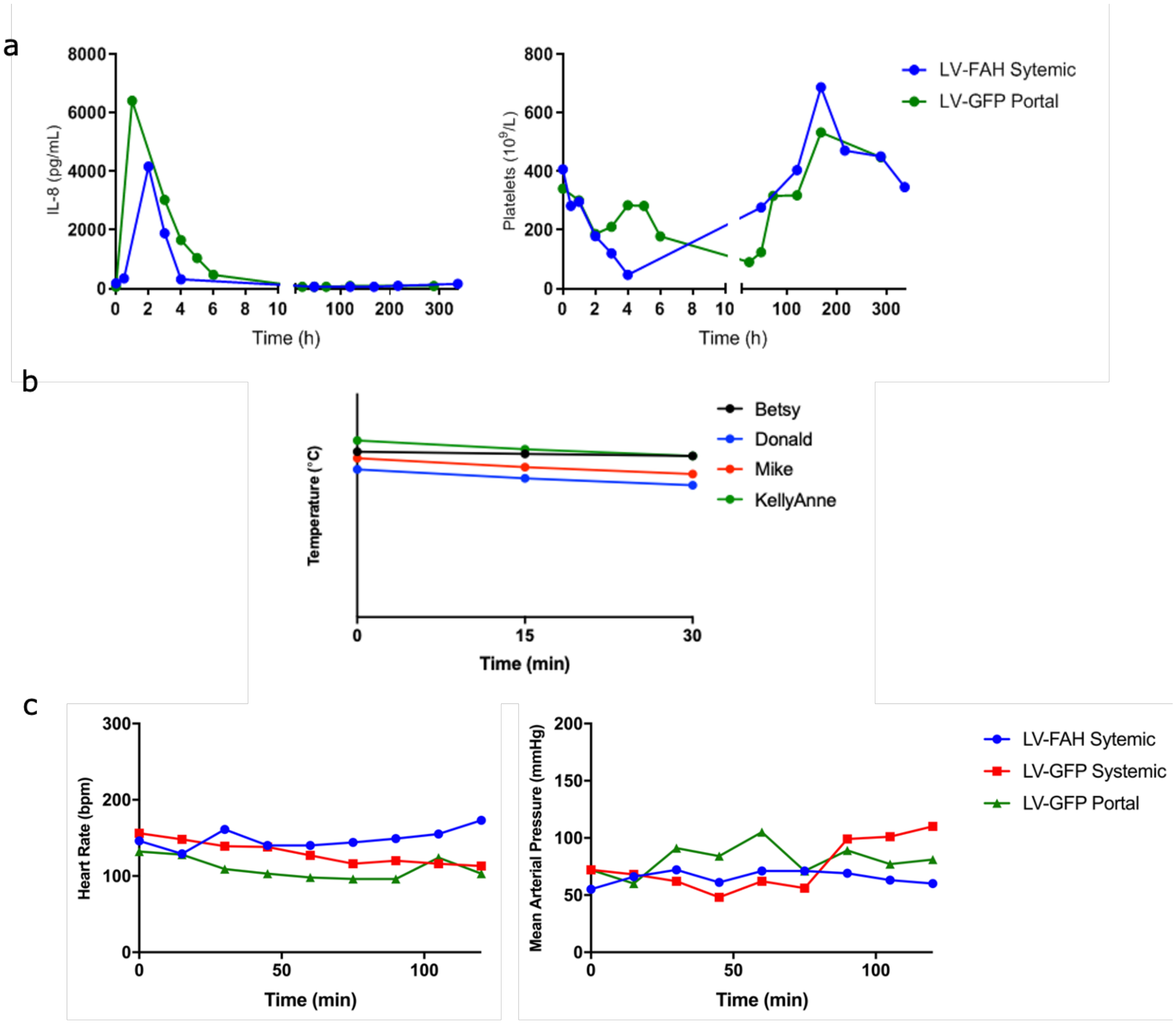

Supplemental Figure 2. H&E staining of myocardium in No. 169 and control. Myocyte hypertrophy and disarray characteristic of hypertrophic cardiomyopathy in pig No. 169 (a) as compared to *FAH*$^{-/-}$ age-matched control (b). Four heart sections were examined with similar results.

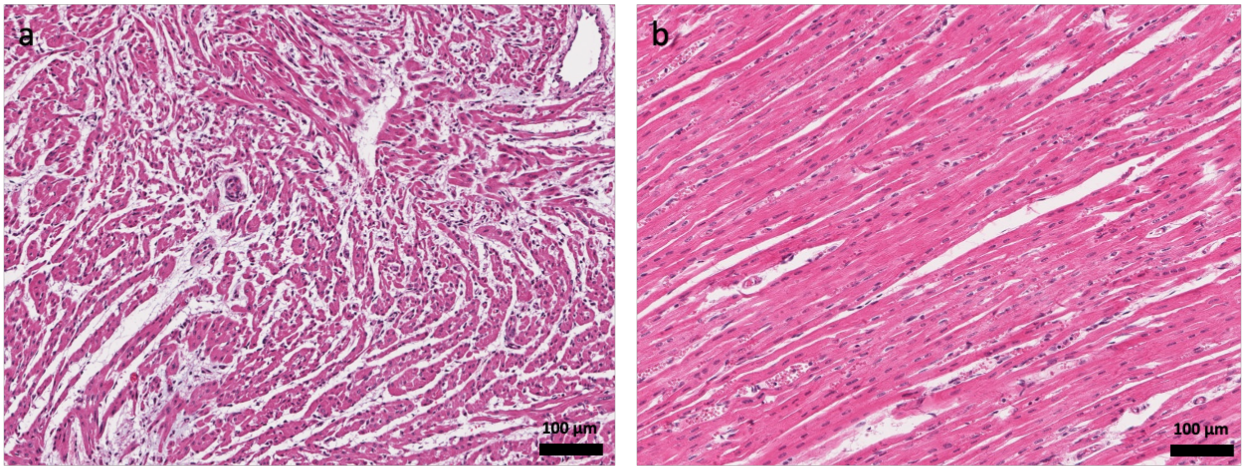

Supplemental Figure 3. Tyrosine levels over time. Day 0 represents pre-treatment blood draw. Day 337 represents post-mortem collection.

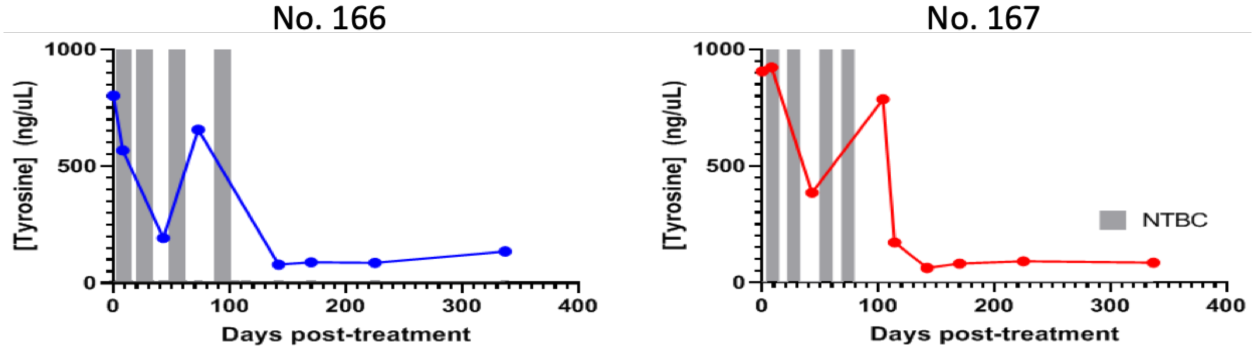

Supplemental Table 1. Summary of blood sample analyses for long term LV-FAH dosed animals. Day 0 represents pre-treatment blood draw. Day 337 represents post-mortem collection.

**Days post-treatment**		**0**	**8**	**43**	**73**	**104**	**114**	**142**	**170**	**225**	**337**
No. 166	ALB	3.1	3	2.3	2.7	-	-	3.2	3.3	3.2	4.2
	ALP	372	321	551	298	-	-	195	180	120	68
	ALT	86	57	99	81	-	-	68	65	48	32
	AST	74	44	37	31	-	-	44	25	19	40
	BILD	<0.1	<0.1	<0.1	<0.1	-	-	<0.1	<0.1	<0.1	<0.1
	BILT	<0.2	<0.2	<0.2	<0.2	-	-	<0.2	<0.2	<0.2	<0.2
	GGT	83	126	126	106	-	-	166	89	66	81
	Ammonia	23	16	49	28	-	-	23	19	59	301
	PT	Clot	10.2	11.7	11	-	-	11.2	10.7	11.1	-
	INR	Clot	0.9	1.1	1	-	-	1	1	1	-
	Phe	57	44	91	74	-	-	68	83	116	203
	Tyr	802	568	193	656	-	-	79	89	86	135
No. 167	ALB	3.7	3.6	3.2	-	2.9	3.5	3.7	3.7	4	3.3
	ALP	307	216	514	-	170	174	135	104	61	24
	ALT	71	72	65	-	67	56	55	40	38	32
	AST	Hem	71	68	-	23	58	35	16	20	81
	BILD	<0.1	<0.1	<0.1	-	<0.1	<0.1	<0.1	<0.1	<0.1	<0.1
	BILT	<0.2	<0.2	<0.2	-	<0.2	<0.2	<0.2	<0.2	<0.2	<0.2
	GGT	70	136	204	-	82	84	82	63	56	125
	Ammonia	16	15	33	-	<10	11	<10	10	38	207
	PT	11.1	10.3	11.7	-	Clot	10.4	11.8	11.1	11.7	9.1
	INR	1	0.9	1.1	-	Clot	0.9	1.1	1	1.1	0.8
	Phe	40	38	121	-	45	55	57	72	92	140
	Tyr	905	923	385	-	786	172	63	81	91	85

"-" indicates sample not tested for that animal occasion.

Supplemental Figure 4. IHC for FAH, H&E, and Trichrome staining in No. 169 at 48h post-treatment. Sixteen liver sections were examined with similar results.

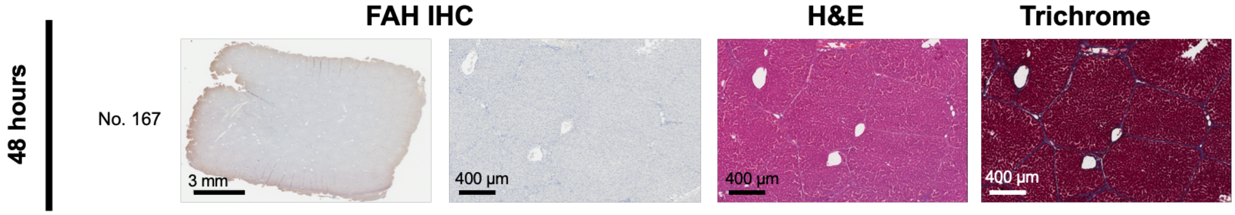

Supplemental Figure 5. High magnification H&E staining and gross liver sections. Restoration of normal hepatic microarchitecture in LV-FAH treated pig (a) as compared to healthy wild-type (b) and $FAH^{-/-}$ sick (c) pigs. Sixteen liver sections were examined with similar results. Gross liver sections from LV-FAH treated pigs 166 (d) and 167 (e) at the time of euthanasia showing no macroscopically visible adenomas. Gross liver sections from $FAH^{-/-}$ sick pig 266 (e) showing multiple adenomas and HCC.

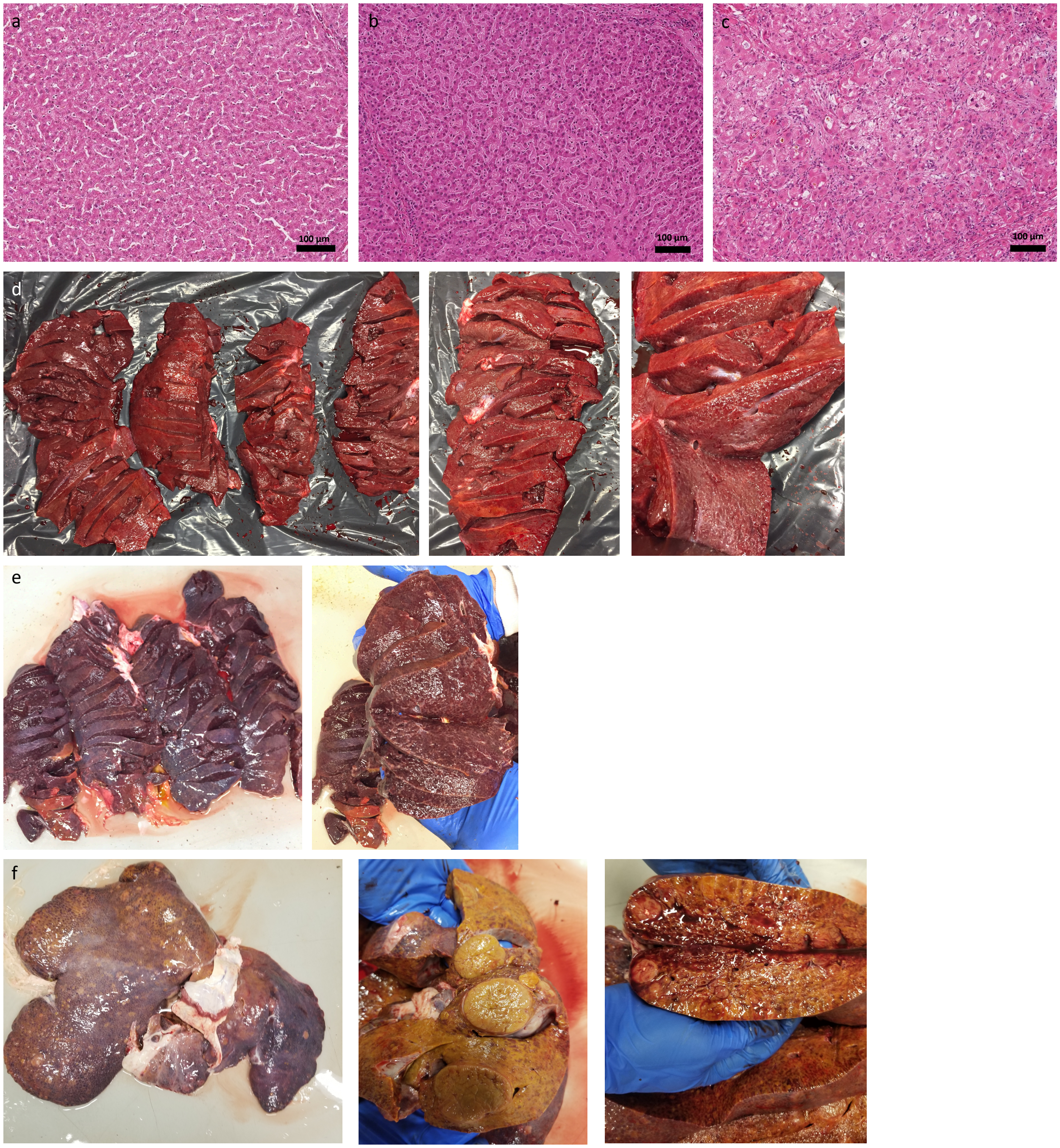

Supplemental Figure 6. Ki-67 and TUNEL staining. (a) Ki-67 and TUNEL staining for experimental animals at 48h, 60 days, 225 days, and 337 days, as well as *FAH*$^{-/-}$ (n=4) and wild-type (n=2) control animals at one year of life. Scale bars represent 400 µm (5x) and 50 µm (40x). (b) Ki-67 and TUNEL staining quantification for experimental animals at 48h (n=4 liver sections, one animal), 60 days (n=2 liver sections, one animal), 225 days (n=4 liver sections, two animals), and 337 days (n=4 liver sections, two animals), as well as *FAH*$^{-/-}$ (n=4) and wild-type (n=2) control animals at one year of life. Y-axis represents positive cells per high powered field. Error bars represent mean and standard deviation. ns represents P>0.05 and ** represents P<0.01 based on a two-sided Welch's t-test with no adjustment for multiple comparisons. Ki67: p=0.009 for 337 day vs *FAH*$^{-/-}$, p=0.007 for wild-type vs *FAH*$^{-/-}$, p=0.18 for 337 day vs wildtype. TUNEL: p=0.06 for 337 day vs *FAH*$^{-/-}$, p=0.1 for wild-type vs *FAH*$^{-/-}$, p=0.5 for 337 day vs wildtype.

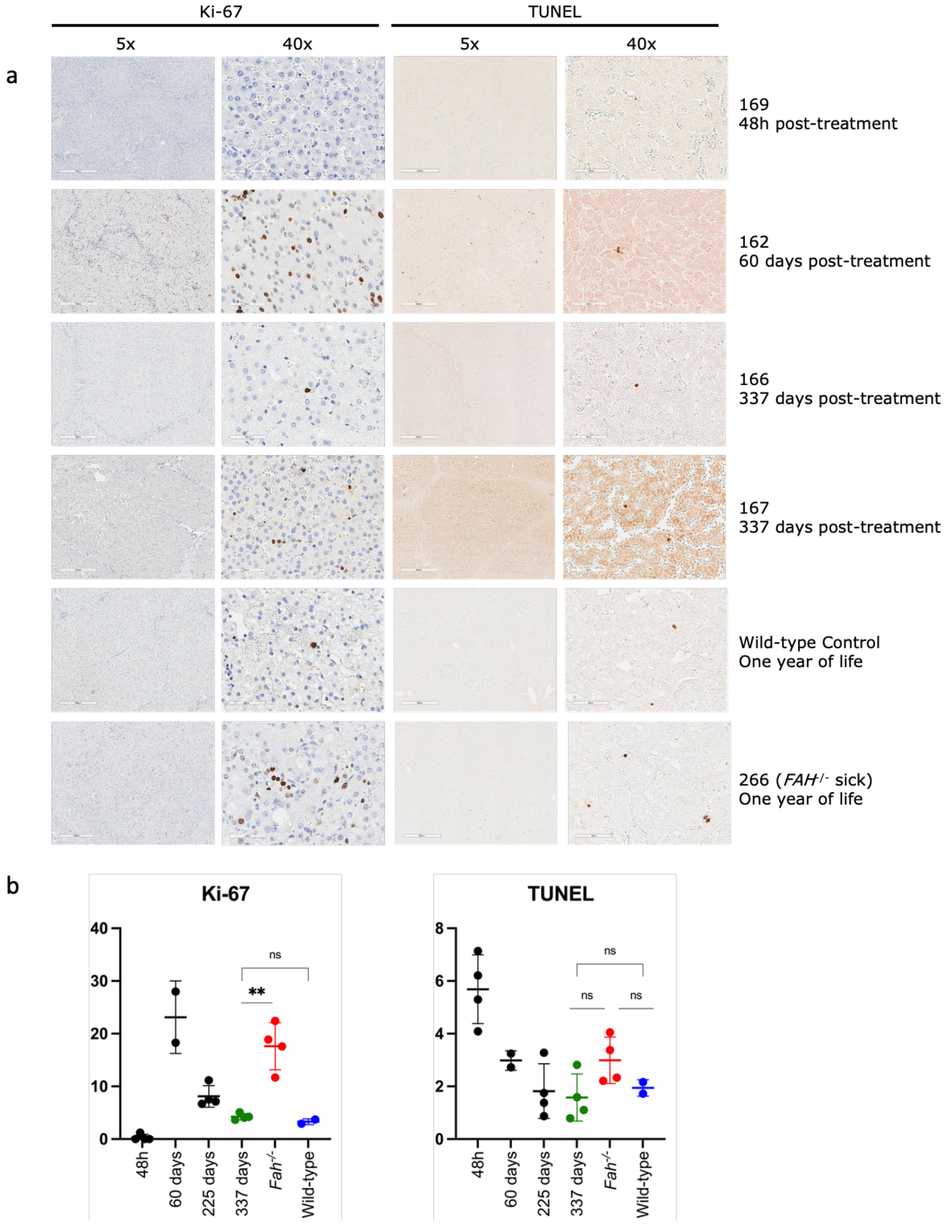

Supplemental Table 2. Percent of fibrotic liver sections per independent pathologist review.

Pig	Time	Site	Sample	% fibrosis	METAVIR
16166 (No. 166)	Day 142	Biopsy 1		60	F2
		Biopsy 2		100	F3
	Day 337	Right Lateral	Anterior Superior	20	F1
			Posterior Superior	20	F1
			Anterior Inferior	30	F1
			Posterior Inferior	30	F1
		Right Medial	Anterior Superior	10	F0
			Posterior Superior	10	F0
			Anterior Inferior	20	F1
			Posterior Inferior	5	F0
		Left Medial	Anterior Superior	2	F0
			Posterior Superior	5	F0
			Anterior Inferior	20	F1
			Posterior Inferior	30	F1
		Left Lateral	Anterior Superior	0	F0
			Posterior Superior	20	F1
			Anterior Inferior	10	F0
			Posterior Inferior	5	F0
16P176 (No. 167)	Day 142	Biopsy 1		50	F2
		Biopsy 2		50	F2
	Day 337	Right Lateral	Anterior Superior	20	F1
			Posterior Superior	5	F0
			Anterior Inferior	10	F0
			Posterior Inferior	20	F1
		Right Medial	Anterior Superior	2	F0
			Posterior Superior	10	F0
			Anterior Inferior	5	F0
			Posterior Inferior	30	F1
		Left Medial	Anterior Superior	2	F0
			Posterior Superior	5	F0
			Anterior Inferior	20	F1
			Posterior Inferior	10	F0
		Left Lateral	Anterior Superior	2	F0
			Posterior Superior	5	F0
			Anterior Inferior	2	F0
			Posterior Inferior	20	F1

Supplemental Table 3. LV-FAH integration mapping statistics.

Percent of Reads with LV-FAH	Total Used Reads for Mapping	Mapped Reads (Total)	Integration Points at >= 1X	Integration Points at >=5X
38.44	55,968,431	637,573 (1.14 %)	17,906	4,425
14.21	66,139,096	14,278,183 (21.59 %)	39,085	8,642
28.54	53,105,410	7,848,599 (14.78 %)	35,298	6,330
